# Inactivation of Wild Poliovirus with β-Propiolactone

**DOI:** 10.3390/vaccines14070642

**Published:** 2026-07-21

**Authors:** Anastasia Kovpak, Sergey Pashkov, Mariia Kostrova, Kirill Nebesnyi, Nikita Yakovlev, Olga Polyakova, Vladislav Vasilenko, Lycheva Natalia, Yury Ivin, Anastasia Piniaeva, Alexandra Siniugina, Aydar Ishmukhametov

**Affiliations:** 1 Chumakov FSC R&D IBP RAS (Institute of Poliomyelitis), Building 1, Village of the Institute of Poliomyelitis 8, Municipal District Filimonkovskiy, Moscow 108819, Russia; pashkov_sa@chumakovs.su (S.P.); kostrova_ma@chumakovs.su (M.K.); nebesnyi_ka@chumakovs.su (K.N.); yakovlev_nv@chumakovs.su (N.Y.); polyakova_os@chumakovs.su (O.P.); vasilenko_ve@chumakovs.su (V.V.); lycheva_na@chumakovs.su (L.N.); ivin_uu@chumakovs.su (Y.I.); pinyaeva_an@chumakovs.su (A.P.);; 2 Institute of Translational Medicine and Biotechnology, Sechenov Moscow State Medical University, Moscow 119991, Russia

**Keywords:** polio, IPV, inactivation, β-propiolactone, viral vaccine, immunization

## Abstract

Objectives: Antiviral vaccines are usually created by inactivating viruses using physical or chemical methods. Inactivation of poliovirus with β-propiolactone (BPL) has advantages, including the absence of a requirement for long-term incubation with the inactivating agent, which minimizes the risk of negative effects of the potentially dangerous substance on the virus and reduces the duration of the inactivation process. Methods: BPL was applied at 0.2% (*w*/*v*) concentration under two conditions: 4 °C for 24 h and 37 °C for 3 h. Inactivation completeness was confirmed on Vero cell culture, while immunogenicity was assessed in guinea pigs via neutralizing antibody test. Conclusions: Two variants of virus inactivation made it possible to obtain inactivated samples that retained their immunogenic properties. BPL-inactivated samples retained sufficient D-antigen levels and elicited neutralizing antibodies comparable to or exceeding those from formaldehyde-inactivated controls. The inactivation method at 37 °C provided faster inactivation, while at 4 °C it provided a smooth decrease in virus titer. These results confirm that β-propiolactone is a viable alternative to formaldehyde for the production of inactivated polio vaccine (IPV), providing rapid inactivation of the virus compared to inactivation with formaldehyde while maintaining immunogenicity, as confirmed by guinea pig control.

## 1. Introduction

Poliomyelitis is a highly contagious disease caused by the poliovirus (*Enterovirus Coxsackiepol*). Poliovirus belongs to the *Picornaviridae* family and is a non-enveloped virus approximately 27 nm, containing infectious RNA. Polioviruses are serologically classified into three types: types 1, 2 or 3 [1]. In 1988, the World Health Assembly adopted the Global Polio Eradication Initiative (GPEI) with a plan to poliomyelitis until 2026. Since wild poliovirus types 2 and 3 have been certified as eradicated, type 3 oral polio vaccines (OPV), like type 2, will also be phased out of the global immunization program [2]. Thanks to immunization campaigns using the oral polio vaccine (OPV), the number of polio cases has significantly decreased. However, OPV is no longer a favorable in terms of the risk of vaccine-derived poliovirus (VDPV) and vaccine-associated paralytic polio (VAPP) cases [3]. A change to an IPV-only vaccination schedule is planned by 2028 [4]. Since wild polioviruses, types 2 and 3 have been certified as eradicated, OPV types 3 (following type 2) will also be excluded from the global immunization program. Thus, the removal of these strains from OPV underscores the importance of producing a safe and affordable IPV [3]. Safe IPV production can be ensured by optimizing the process of virus inactivation using various methods. Formaldehyde is a classic inactivating agent that has been used in the production of inactivated polio vaccines since 1954. The process of poliovirus inactivation with formaldehyde (final concentration 0.025%, m/v) takes 13 days at a temperature of 37 °C [5]. The inactivation of viruses with formaldehyde has several drawbacks, the most important of which is its negative impact on the virus’s antigenic structure [3]. Such conditions lead to thermal degradation of the virus and destruction of viral epitopes due to prolonged exposure to elevated temperatures [6]. Therefore, it is important to select inactivating agents that are easy to use and allow the preservation of a native structure of viral epitopes. Modern inactivating agents suitable for the inactivation of the poliovirus include hydrogen peroxide, aascorbic acid, epigallocatechin-3-gallate [3] and gamma radiation method [7].

One of common inactivating agent is β-propiolactone (BPL), an organic compound widely used for virus inactivation. BPL has been used in the development [8] and manufacturing of inactivated vaccines against influenza [9], SARS-CoV [10,11] and SARS-CoV-2 [12,13,14,15,16]. The mechanism of BPL inactivation is based on the irreversible alkylation of nucleic acids. Because of these reactions, BPL form adducts with nucleic acid bases, mainly guanine [17,18], and induces extensive modifications of nucleic acid base analogs, nucleosides, and synthetic peptides [19]. Successful inactivation of pathogens, while preserving the required structure of BPL-inactivated samples, depends on a combination of various influencing factors: BPL concentration, stable pH during inactivation, temperature, inactivation time, and the stability of the pathogen itself [20]. In addition, it is necessary to carry out the stage of hydrolyzing residual BPL, which allows the toxic and carcinogenic BPL to decompose into beta-hydroxypropionic acid [19]. The use of BPL can make the production safer by inactivation process at the beginning of the production cycle. Carrying out inactivation in this way will make it possible to obtain a vaccine purified from the residual content of BPL and/or its products already at the first stage of chromatographic purification (exclusion chromatography), which eliminates the need to include BPL in the formulation vaccine. Available publications on poliovirus inactivation using BPL refer to experiments carried out more than 40 years ago and vary significantly in their methodology [21]. Therefore, the aim of our study was to investigate the ability of BPL to completely inactivate poliovirus without the formation of carcinogenic products during its degradation, and to evaluate its ability to induce neutralizing antibodies in guinea pigs.

## 2. Materials and Methods

### 2.1. Cells

A Vero cell culture was used for poliovirus propagation (Chumakov FSC R&D IBP RAS (Institute of Poliomyelitis), Moscow, Russia). For inactivation efficiency control, two sensitive cell cultures, Vero and HEp-2 (Cincinnati) (Chumakov FSC R&D IBP RAS (Institute of Poliomyelitis), Moscow, Russia), were used. To determine the inactivation curve, infectivity titers and neutralization reaction, HEp-2 (Cincinnati) cell culture was used.

### 2.2. Viruses Strains

The study used wild poliovirus strains Mahoney (type 1) and Saukett (type 3), and Sabin strains type 1 and 3 from the collection of the Chumakov FSC R&D IBP RAS (Institute of Poliomyelitis, Moscow, Russia).

### 2.3. Animals

Guinea pigs (*N* = 30) of the both sexes (the same number of females and males) weighing 250–300 g were used to evaluate the immunogenic activity of inactivated poliovirus samples (Mahoney and Saukett strains). Animals were purchased from Scientific Center of Biomedical Technologies, branches Andreevka or Stolbovaya, Russia. They were maintained under standard conditions in accordance with the Guide for the Care and Use of Laboratory Animals [22] and Directive 2010/63/EU [23]. All procedures involving animals were approved by the Bioethics Committee of the P.V. Sechenov Institute of Research and Development, RAS (Polio Institute) (#141025-3 from 14 October 2025).

### 2.4. Preparation of Cell and Viral Suspension

Vero cells were expanded in EMEM nutrient medium (Chumakov FSC R&D IBP RAS (Institute of Poliomyelitis), Moscow, Russia) supplemented with 10% fetal bovine serum (Research Grade, Wellington, New Zealand) and 1.5 g of L-glutamine (Chumakov FSC R&D IBP RAS (Institute of Poliomyelitis). Moscow, Russia) in a ReadyToProcess Wave 25 wave-type bioreactor (Cytiva, Marlborough, MA, USA) using microcarriers (4 g/L) LXMC-Dex1 (SunResin, Xi’an, China) in pseudo-suspension mode (culture conditions: 37 °C; pH 7.2 ± 0.1; DO 70%). The seeding cell concentration for bioreactor inoculation was 0.20 ± 0.05 × 10^6^ cells/mL. Upon reaching a dense monolayer on microcarrier particles, the cells were infected. Before infection, microcarriers were sedimented and washed with Hank’s solution (Chumakov FSC R&D IBP RAS (Institute of Poliomyelitis), Moscow, Russia). Virus and medium 199 (Chumakov FSC R&D IBP RAS (Institute of Poliomyelitis), Moscow, Russia) were added to the bioreactor. Virus culture conditions: 37 °C; pH 7.2 ± 0.1; DO 70%.

### 2.5. Virus Inactivation with BPL

In this work, we were based on the most common conditions for virus inactivation using BPL, which were previously mentioned by other researchers. Table 1 shows the conditions for virus inactivation using BPL.

Development of the poliovirus inactivation process we used methods, based on previously described procedures, with minor modifications, regarding the incubation time of samples with BPL and neutralization of residual BPL. For each inactivation method, four liters of viral suspension were used, one liter each from four collections from the bioreactor. The titer of viral suspension for Mahoney and Saukett was 6.87 ± 0.5 and 7.25 ± 0.5 TCID 50/mL (50% tissue culture infectious dose) respectively. For BPL inactivation, we used undiluted BPL (Acros Organics, Waltham, MA, USA). Inactivation was performed in spinner flasks (Corning, New York, NY, USA). pH was controlled with sodium bicarbonate (Chumakov FSC R&D IBP RAS (Institute of Poliomyelitis), Moscow, Russia) and determined using pH indicator strips (Merck Millipore, Burlington, MA, USA). The virus inactivation experiment was performed in triplicate for each inactivation method. We used two methods of inactivation:

- Method 1: BPL was added to a viral suspension (the volume of the virus was 1 L), preheated to 37 °C at a final concentration of 0.2% (*w*/*v*). The mixture was stirred and the pH was adjusted to 7.4–7.6. The contents were then transferred to a spinner flask and incubated with constant stirring at 37 °C for 3 h (BPL-37). This procedure was repeated for three other virus collections.
- Method 2: BPL was added to the viral suspension at a concentration of 0.2% (*w*/*v*) and mixed, and the pH was adjusted to 7.4–7.6. The mixture was then transferred to a new flask and incubated with constant stirring at a temperature of 4 °C for 24 h. Six hours after the start of inactivation, suspension was filtered into a new flask through 0.22 µm filter (Corning). Following inactivation, the samples were additionally incubated at 37 °C for 3 h without stirring (BPL-4). This procedure was repeated for three other virus collections.

Thus, at the end of the inactivation process, twelve series of inactivated viral suspension (three liters by method 1 and three by method 2 for each strain) were obtained one liter each. After determining the residual infectivity, suspensions obtained at the same inactivation temperatures were combined and approximately 3 L of inactivated virus of each type were obtained (Table 2).

### 2.6. Virus Inactivation with Formaldehyde

According to the IPV production technology [29] inactivation of the viral suspension (approximated 1 L for each wild-type of poliovirus with titer 6.75 and 7.0 TCID 50/mL for Mahoney and Saukett respectively) with formaldehyde was carried out after filtration (0.22 µm (PES)), ultrafiltration (100 kDa, PES) and chromatographic purification steps as the previously described [29,30]. Briefly, formaldehyde was added (drop by drop) to the purified viral concentrate (approximated volume 30 mL) to a final concentration of 0.025% and incubated for 13 days at a temperature of 37 °C with constant stirring. This procedure was repeated once for two types of poliovirus.

### 2.7. Residual BPL and Formaldehyde Neutralization

Residual BPL and formaldehyde was neutralized with sodium thiosulfate (2% (*w*/*v*)) (OOO “DALHIMPHARM”, Moscow, Russia) and sodium sulfite 0.0264 M (Sigma, Ronkonkoma, NY, USA) respectively.

### 2.8. Preparation of Inactivated Viral Concentrates

Suspensions obtained at the same inactivation temperatures were combined and approximately 3 L of inactivated virus of each type was obtained (Table 2). To test the immunogenic activity of BPL-inactivated polioviruses, viral samples were subjected to purification host cell DNA (hcDNA), BSA and host cell proteins. BPL-inactivated viral suspension were clarified using a filter cascade (0.45/0.2 µm (PES)) and concentrated using tangential flow filtration method (100 kDa, PES). The obtained viral concentrates were purified from cellular and serum proteins, as well as hcDNA, using two-step chromatography: gel filtration [29] and ion-exchange chromatography as previously described [30].

### 2.9. Detection of Live Virus After Inactivation

Confirmation of virus inactivation was performed by cell culture. Vero and HEp-2 (Cincinnati) cells with a dense, formed monolayer in 25 cm^2^ plastic culture flasks (Corning, USA) were used. Each inactivated sample was filtered via 0.22 µm sterile syringe filters (Corning, New York, NY, USA) and inoculated into two flasks (each flask contained 2 mL sample and 8 mL EMEM medium with a ratio of 1:4). Flasks were incubated for 7 days at 37 °C. Complete inactivation was confirmed through blind passage two times using the same method. Flasks were examined under a microscope for the presence of cytopathic effect (CPE).

### 2.10. Determination of D-Antigen by Enzyme-Linked Immunosorbent Assay (ELISA)

Poliovirus D-antigen content was determined by ELISA in a sandwich format, developed at Chumakov FSC R&D IBP RAS (Institute of Poliomyelitis, Moscow, Russia) [31].

### 2.11. Determination of the Infectivity Titers of Polioviruses

Polioviruses were titrated using a standard method, where each sample was serially diluted 10-fold in EMEM medium and inoculated onto HEp-2 (Cincinnati) cells in 96-well culture plates (Corning, New York, NY, USA). Plates were incubated for 7 days with daily microscopic examination for the presence of cytopathic effect (CPE). The infectivity viral titer in log TCID_50_/mL was calculated by the Kärber formula [3].

### 2.12. Inactivation Kinetics

The inactivation time of viruses takes from 6 to 72 h (Table 1) at various concentrations of BPL. In our work, we used two-time intervals: for a temperature of 4 °C—24 h and for 37 °C—3 h. To determine the time of complete inactivation of the virus, the kinetics of inactivation was determined: aliquots were taken from each spinner flask containing 1 L of viral suspension at time intervals throughout the process. For 24 h virus inactivation: 0, 1, 2, 3, 4, 5, 6, 7, 8, 24 h; for 3 h virus inactivation: 0, 0.5, 1, 1.5, 2, 2.5, 3 h. Results are presented as mean values of virus infectivity titers expressed in log TCID_50_/mL [3].

### 2.13. Immunization of Guinea Pigs and Neutralizing Antibody Titration

Five animals were used for testing each sample. Animals were randomized by weight between groups:

- Mahoney BPL, 4 °C, 24 h (*N* = 5).
- Mahoney BPL, 37 °C, 3 h (*N* = 5).
- Mahoney formaldehyde, 37 °C, 13 days (*N* = 5).
- Saukett BPL, 4 °C, 24 h (*N* = 5).
- Saukett BPL, 37 °C, 3 h (*N* = 5).
- Saukett formaldehyde, 37 °C, 13 days (*N* = 5).

The inactivated samples, purified by chromatography in phosphate-buffered saline, were administered to animals without the addition of adjuvants. Guinea pigs were immunized intramuscularly with a single 0.5 mL of solution per animal, 0.25 mL in each pelvic limb and blood was collected on day 7. After centrifugation at 2000× *g* for 12 min, serum samples were heat-inactivated at 56 °C and tested in a neutralization assay. The presence of virus-neutralizing antibodies in serum samples was determined using a neutralization test (NT) in HEp-2 cells (Cincinnati) against attenuated Sabin strains (types 1 and 3) and wild-type strains (Mahoney and Saukett), according to the standard method [32].

### 2.14. Statistical Data Processing

Descriptive statistics using Microsoft Office Excel 2013 software (Microsoft, Redmond, WA, USA) were used to present quantitative data obtained during virus inactivation kinetics and neutralizing antibody titer determination. The number of experiment repeats (*n*) is indicated for each experiment. One-way analysis of variance (ANOVA) test was used for data comparison. Differences were determined at a significance level of *p* < 0.05. Statistical analysis was performed using GraphPad Prism 8.0.1 (Boston, MA, USA).

### 2.15. Biosafety and Biosecurity Measures

Chumakov FSC R&D IBP RAS (Institute of Poliomyelitis, Moscow, Russia) is an OPV producer and scientific research institute. It is fully accredited by the national authorities for work with BSL 1–3 agents and it has a Certificate of participation in poliovirus containment (RUS-CP-20191202-007) issued by GCC.

## 3. Results

### 3.1. Inactivation

Virus inactivation was developed using samples obtained under conditions close to real IPV production on microcarriers, which entails the presence of various in the test samples, such as cell and serum proteins, hcDNA and microcarrier particles. Such conditions represent an additional burden on the inactivator and require thorough verification of inactivation efficiency. Viral suspensions of the Mahoney and Saukett strains have been received with titers of 6.87 ± 0.5 and 7.25 ± 0.5 TCID 50/mL, respectively.

### 3.2. Inactivation Kinetics

**Figure 1:**
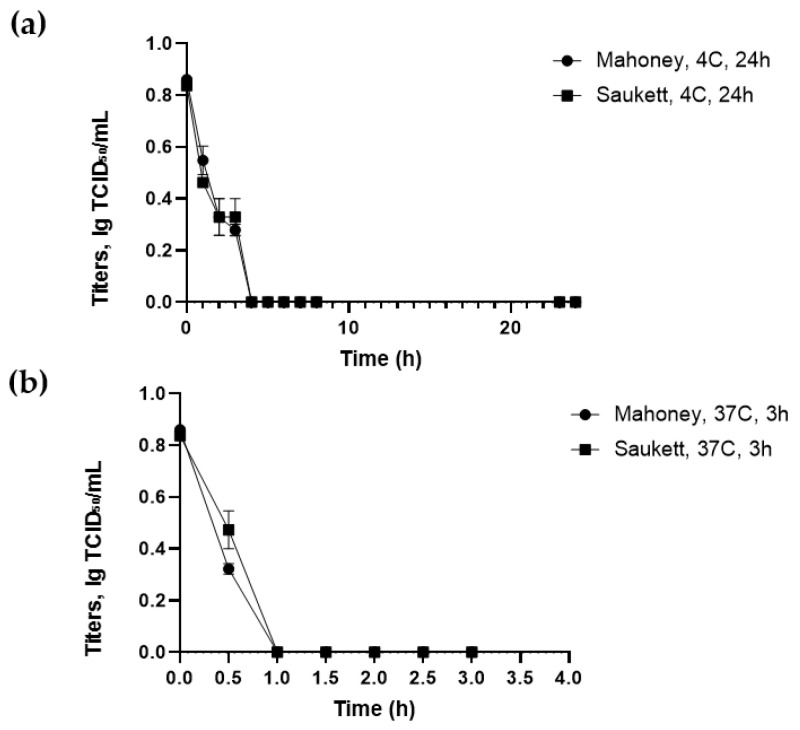
Inactivation kinetics of Mahoney and Saukett strains at (**a**) 4 °C for 24 h, (**b**) 37 °C for 3 h (*n* = 3).

BPL has shown its ability to inactivate wild poliovirus strains in a short time compared to the traditional method of inactivating polioviruses using formaldehyde. CPE was no longer detectable after 1 h in the case of inactivation method 1, and 4 h in the case of method 2. Figure 1 shows the inactivation kinetics of viral suspensions of Mahoney and Saukett strains inactivated using BPL.

Based on the inactivation kinetics results, it can be concluded that at 37 °C, virus inactivation occurs faster. Half an hour after the start of inactivation, the titer of the virus decreases by more than 50% (68.7 ± 4.5% for Mahoney strain and 56.2 ± 8% for Saukett), and as early as 1 h after the start of inactivation, no live virus is detected in the test samples by titration. Inactivation of viruses at 4 °C occurs more gradually and does not cause such a rapid drop in virus titer as at 37 °C (Figure 1b), which indicates the absence of additional thermal inactivation and likely less negative impact on the virus. A noticeable decrease in the titer of the virus occurs 4 h after the start of inactivation, and its decrease is 73.8 ± 0.4% and 68.8 ± 4.5% for Mahoney and Saukett strain, respectively. In this case, in-process filtration was used to prevent the formation of viral and/or protein aggregates that could affect the inactivation process and lead to incomplete poliovirus inactivation. Such filtration does not significantly affect the viral particle or antigen content, since sterile filtration (0.22 μm) is already employed as the first purification step in IPV manufacturing and has been shown to have no detrimental effect on these parameters [29].

### 3.3. Inactivation Efficiency

**Figure 2:**
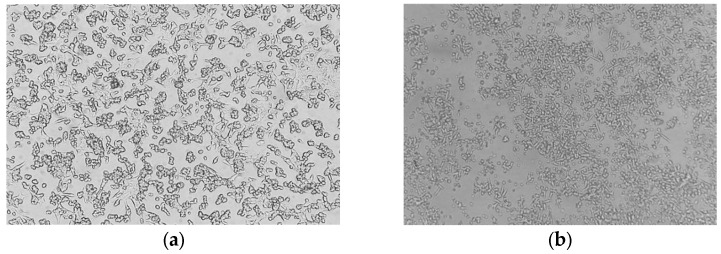
Appearance of Vero (**a**) and HEp-2 (Cincinnati) (**b**) cells subjected to toxic effects of the inactivator. Magnification ×100.

**Figure 3:**
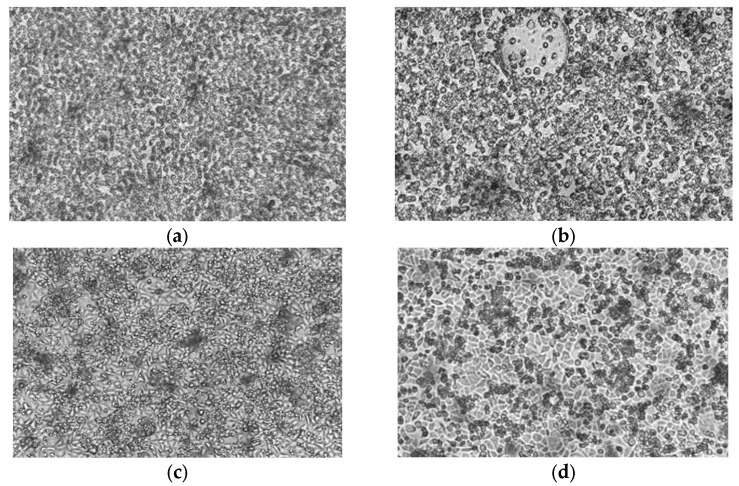
The second passage of residual infectivity control on HEp-2 cell: (**a**) negative control (uninfected cells), (**b**) positive control (viral CPE), (**c**) cells infected with inactivated Mahoney strain at 37 °C, (**d**) cells infected with inactivated Saukett strain at 37 °C. Magnification ×100.

**Figure 4:**
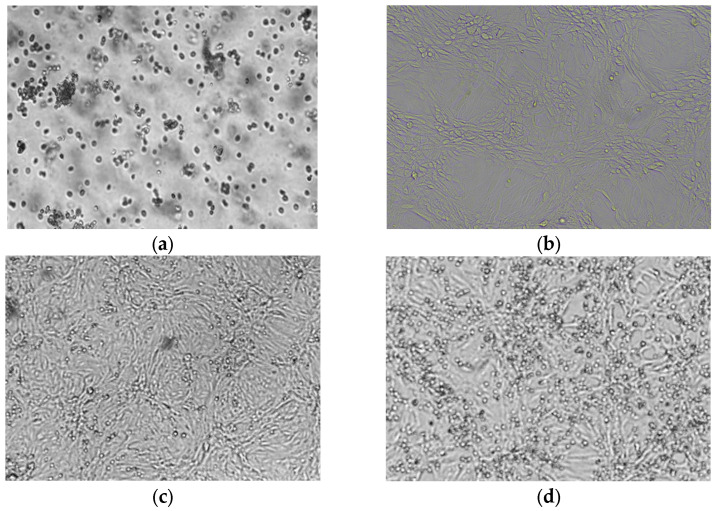
The second passage of residual infectivity control inactivated Mahoney strain: (**a**) positive control (viral CPE), (**b**) negative control (uninfected cells), (**c**) cells infected with inactivated virus at 4 °C, (**d**) cells infected with inactivated virus at 37 °C.

**Figure 5:**
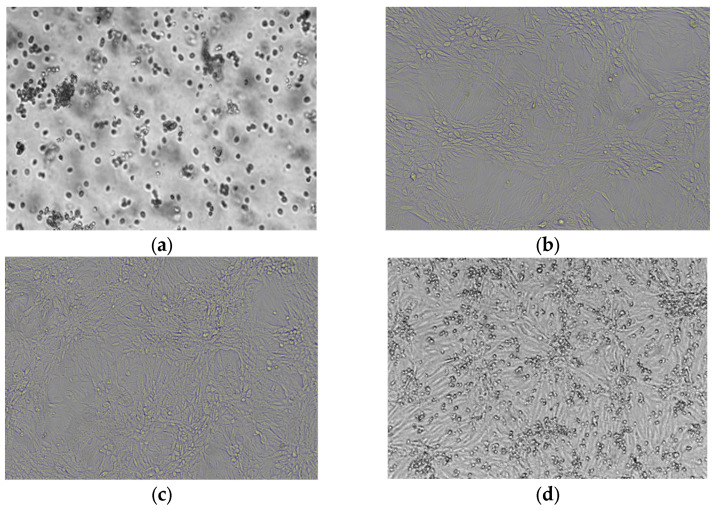
The second passage of residual infectivity control inactivated Saukett strain: (**a**) positive control (viral CPE), (**b**) negative control (uninfected cells), (**c**) cells infected with inactivated virus at 4 °C, (**d**) cells infected with inactivated virus at 37 °C.

After inactivation, the samples were tested for residual poliovirus. It has been shown that during control of residual infectivity in the first passage, both Vero and HEp-2 (Cincinnati) cells can experience degenerative changes in the cellular monolayer-caused cell death that were not associated with viral replication (Figure 2). This phenomenon may be a consequence of the toxic effect on the cellular monolayer of decay products of BPL and/or sodium thiosulfate, which was used to neutralize the residual BPL in the samples. In the second passage, this phenomenon was not observed due to dilution of the sample. It is important to note that, despite the sensitivity of the HEp-2 (Cincinnati) cell culture it is not suitable for this type of control, since the cells grow rapidly, forming additional layers on live cells (Figure 3c,d), the same as in the control (Figure 3a) which makes it difficult to objectively visually evaluate the experiment. Therefore, in this work, the conclusion about the successful control was taken into account and the results of monitoring residual infectivity only on Vero cell culture. When monitoring residual infectivity using Vero cell culture, a monolayer of healthy cells is clearly visible in the second passage (Figure 4c,d and Figure 5c,d), which allows us to conclude that the poliovirus is completely inactivated with BPL.

### 3.4. Immunogenic Activity

The samples were tested for the content of the target antigen (D-antigen) after inactivation and chromatographic purification (Table 3).

As presented in Table 3, content of D-antigen in samples inactivated with BPL and formaldehyde differs. This is due to the different initial volume of the viral suspension of each type of virus used in experiments. Despite differences in target antigen content, before animal immunization, the D-antigen concentration (DU/mL) in the test samples was equalized and amounted to 12 DU/mL for the Mahoney strain and 18 DU/mL for the Saukett strain. Inactivated samples used for immunization contained 6 DU/dose of antigen for Mahoney strain and 9 DU/dose for Saukett strain.

Blood serums obtained from animals after immunization with the studied samples were tested in a neutralization test (Table 4) against wild poliovirus strains and attenuated Sabin strains with titers of 10.0 ± 0.5 and 9.7 ± 0.5 TCID 50/mL for type 1 and 3, respectively.

**Figure 6:**
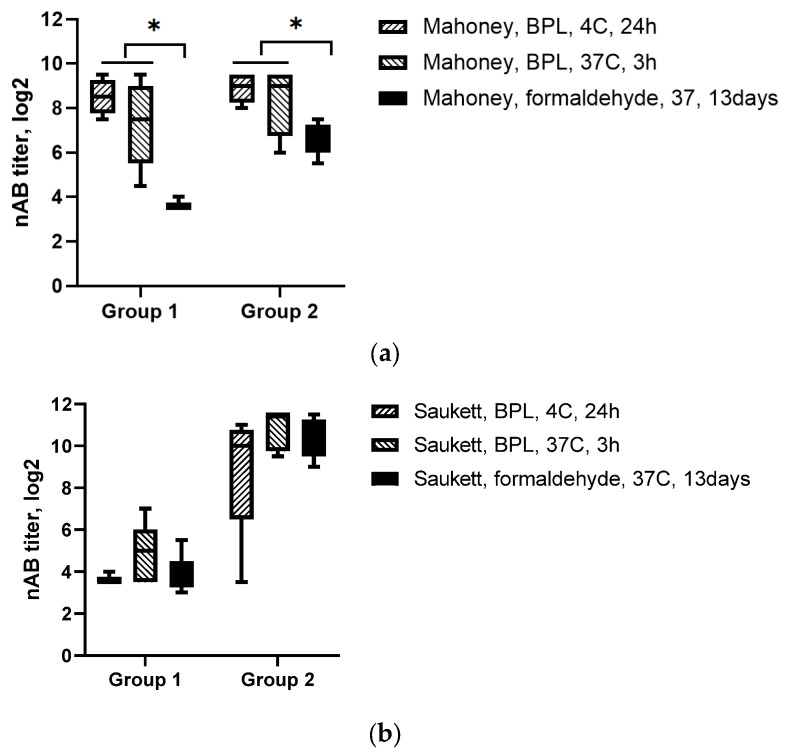
nAB titers inactivated samples against wild polioviruses (group 1) and vaccine Sabin strains (group 2): (**a**) inactivated Mahoney strains, (**b**) inactivated Saukett strains. The differences between values of nAB titer-inactivated Mahoney strain with BPL and formaldehyde are statistically significant (one-way ANOVA test, * *p* < 0.05).

The titers comparison of neutralizing antibodies obtained in the neutralization reaction against the wild (group 1) and Sabin strains (group 2) type 3 are shown in Figure 6a. When using BPL to inactivate poliovirus of the Mahoney strain (by method 1 and 2), these nAB titers differ from inactivation with formaldehyde. Figure 6b also shows a comparison of the titers of neutralizing antibodies obtained in the neutralization reaction against wild (group 1) and Sabin strains (group 2) type 3. We did not observe any differences in the titers of neutralizing antibodies obtained after immunization of animal viruses with inactivated formaldehyde and BPL (Figure 6b). It can also be noted that the titers of antibodies during neutralization against the wild strain and the Sabin strain of antibodies do not have significant differences regardless of the method of inactivation using BPL. However, the levels of antibodies produced by the Mahoney and Saukett strains are different (Table 4), which can be explained by differences in the induction of nAB in different types of polioviruses and the different effects of the inactivating agent on the viral particle.

## 4. Discussion

It takes 13 days to inactivate the poliovirus with formaldehyde. In our work, we have shown that the poliovirus of the Mahoney and Saukett strains can be inactivated with BPL in less than 8 h at a temperature of 4 °C and 3 h at a temperature of 37 °C. (Figure 1). Such an inactivation method takes less time compared to inactivation with formaldehyde, which confirms the advantages of using BPL. It was previously shown that the BPL inactivation mechanism is based primarily on carboxyethylation of guanine and adenine, which does not significantly affect the target viral antigens [33]. However, BPL is capable of interacting with viral capsid proteins, which may be determined by, for example, the temperature at which inactivation is carried out [34]. Therefore, BPL inactivation is often carried out at 4 °C due to its instability at temperatures exceeding this [35]. However, conducting the inactivation process with BPL at 4 °C slows down BPL hydrolysis [36] and leads to prolonged interaction with the antigenic epitopes of the virion and viral RNA, which may result in reduced viral antigen content. During inactivation, the pH of the inactivation medium also influences the nature of the viral proteins that interact with BPL. Furthermore, the nature of the proteins that interact with BPL also changes when the pH becomes more acidic [37]. Despite adjusting the pH during inactivation to 7.6, its decrease may also lead to a change in the confirmation of the surface proteins of the virus, may enhance the interaction between the BPL and viral proteins and, consequently, reduce immunogenicity.

The inactivation stage is critical in the IPV production process, and testing samples for residual live poliovirus after the inactivation process is necessary. A common accepted method for determining residual infectivity of poliovirus in vaccine is the use of blind passages in cell culture of HEp-2 [3,21]. However, this method may cause difficulties in detecting of residual virus CPE, since according to the standard method, the dilution of the studied samples in a nutrient medium is 1:4 (*v*/*v*), which requires a long time of monitoring the cell culture. The test was performed on two permissive cell culture, followed by two consecutive subcultures, and these subcultures were monitored for the maximum technically possible period of time (7 days) to allow any traces of the live virus to cause visible CPE (Figure 4c,d and Figure 5c,d). Although this test confirmed the complete inactivation of all inactivated viral suspensions, there is a need to develop additional methods to control residual infectivity. Such a method could be qPCR, a method that demonstrates the decay of the viral genome during inactivation and indicates the inability of the virus to replicate further. However, the data obtained earlier [38] indicate that the use of this method is difficult, since parts of the viral genome are susceptible to degradation in different ways. It has been shown that some areas of the genome are more resistant to BPL inactivation, while others are highly susceptible to degradation. Therefore, the choice of the genome segment to be amplified is of paramount importance for obtaining reliable results. According to the data obtained earlier, this study used a generally accepted method used in the production of the IPV.

Another important parameter for quality control of the IPV is the evaluation of the immunogenicity of the vaccine. Based on the neutralization test results, a conclusion can be drawn about the immunogenicity of BPL-inactivated samples. Previous studies have shown that the level of neutralizing antibodies must be higher than 1:8 to be considered protective [39]. In our study, titers of at least 1:8 were obtained for all methods of virus inactivation with BPL (Table 4), which indicates the immunogenicity of the samples obtained. The dose of antigen during animal immunization was 12 and 18 DU/mL for the Mahoney and Saukett strains, respectively. We observed differences in neutralization titers against the wild strain and the Sabin strain only for inactivated BPL samples of the Mahoney strain (Table 4). It can be explained by the antigenic differences between the strains [40] and their neutralizing ability. Similar differences in the level of neutralizing antibodies to different strains can also be observed in weakened Sabine strains inactivated with formaldehyde [5].

## 5. Conclusions

Our work demonstrates that inactivation of wild strains of Mahoney and Saukett poliovirus with BPL at concentration of 0.2% allows us to obtain fully inactivated viruses after four hours at a temperature of 4 °C and an hour at a temperature of 37 °C. The results obtained are confirmed by the control of residual infectivity on Vero cell culture. Inactivated and then purified viral suspensions induce protective titers of neutralizing antibodies in immunized animals against wild and weakened strains (Table 2). The developed inactivation methods can be used in the production of IPV as an alternative to the standard inactivating agent formaldehyde, the inactivation stage of which takes 13 days. Unlike formaldehyde, which is part of polio vaccines and the content of which must be monitored, inactivation with BPL as the first stage of vaccine production makes it possible to obtain a product free of this impurity at the downstream stages and, accordingly, does not require the inclusion of a potentially carcinogenic substance into the vaccine.

## Figures and Tables

**Table 1:** Conditions of viruses inactivation with BPL.

Time, h	Concentration of BPL, %	Temperature, °C	Virus
24	0.025	4	SFTS [24]
18	0.1, 0.2, 0.4	4	Poliovirus [21]
24	0.1	4	Influenza [8]
72	0.05, 0.03, 0.025	4	DTMUV [25]
24	0.02	4	VHSV [26]
24	0.025	4	Rabies virus [27]
6	0.5, 0.25, 0.15	37	Influenza [28]

**Table 2:** Characteristics of inactivated viral suspensions.

Virus Type	Inactivation Method	Approximated Volume, L
Mahoney	BPL-4	3.0
Mahoney	BPL-37	3.0
Saukett	BPL-4	3.0
Saukett	BPL-37	3.0
Mahoney	Formaldehyde	1.0
Saukett	Formaldehyde	1.0

**Table 3:** Content of D-Antigen in inactivated samples by ELISA.

Inactivation Conditions	Strains	D-Antigen, DU/mL
BPL, 4 °C, 24 h	Mahoney	16.1 ± 5.1
BPL, 37 °C, 3 h		12.2 ± 1.2
Formaldehyde, 37 °C, 13 days		30.5 ± 2.1
BPL, 4 °C, 24 h	Saukett	18.1 ± 3.6
BPL, 37 °C, 3 h		25.7 ± 4.2
Formaldehyde, 37 °C, 13 days		31.8 ± 1.1

**Table 4:** Neutralization reaction results of blood sera.

Virus Type	Inactivator	nAB Titers log2 (Sabin)	GMT, 95% CI (Sabin)	nAB Titers log2 (Wild)	GMT, 95% CI (Wild)
Mahoney	BPL-4	8.5 ± 0.7	1:375 (138.09–706.71)	8.9 ± 0.6	1:494 (253.29–821.91)
	BPL-37	7.1 ± 1.9	1:167 (77.25–662.85)	8.3 ± 1.5	1:326 (57.94–863.66)
	Formaldehyde	3.5 ± 0.2	1:12 (-)	6.6 ± 0.7	1:101 (46.11–177.89)
Saukett	BPL-4	3.5 ± 0.2	1:12 (-)	8.2 ± 3.1	1:494 (118.61–2036.59)
	BPL-37	4.6 ± 1.4	1:28 (-)	10.7 ± 0.9	1:1465 (1024–2048)
	Formaldehyde	3.7 ± 0.9	1:14 (-)	10.3 ± 0.9	1:1110 (512–1953.65)

nAB—neutralizing antibody, antibody data are presented as mean ± standard deviation, GMT—geometric mean titers, CI—confidence interval.

## Data Availability

The data presented in this study are contained within the article.
